# Supplementary material for: Studying Parameters Affecting Accumulation of Chilling Units Required for Olive Winter Flower Induction
Source: Plants (Basel). 2023 Apr 20;12(8):1714. doi: 10.3390/plants12081714 (PMC10143890; doi:10.3390/plants12081714)
Supplement: Supplementary file 1 [file plants-12-01714-s001.zip › plants-2279901-supplementary.pdf]

Supplementary Figure S1. Number of buds per branch (*n*) and percent buds forming inflorescences (*i*) under different levels of fruit load. Experiment was performed on four potted fruit-bearing 'Barnea' trees. Potted trees were transferred to a controlled environment of 16/10 °C (day/night) on July 27th, 2020 until October 11th, 2020. Branches with or without (w/o) fruit (ten each) were marked at the beginning of the experiment. Average *n* values (A) and *i* values(B) evaluated on November 2020 are presented. The error bars represent the standard error of the mean for each treatment.

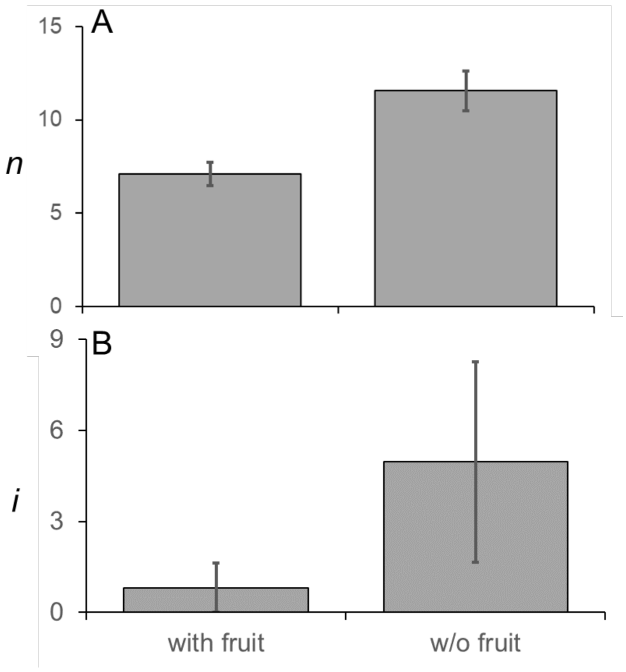

Supplementary Figure S2: Changes in branch water potential (MPA) under forced drought (FD) treatment. Water potential values are in megapascals (MPa) calculated from one branch per plant, 5 biological repeats (plants) per cultivar per treatment (well-watered and FD) throughout the experimental drought period. There were six sampling dates, one week apart, starting two weeks after the FD treatment began. Values for each tree in the experiment are presented. An asterisk marks drought plants irrigated for a single day (one asterisk) or a few days (two asterisks connected by a dotted line). All drought treated plants received irrigation between the 24th and 27th of December (Green asterisk) A-B. 'Picholine', C-D. 'Barnea', E-F. 'Souri', G-H. 'Coratina', I-J. 'Koroneiki'. Numbers in Coratina Forced drought treatment are *i* values for each of the trees.

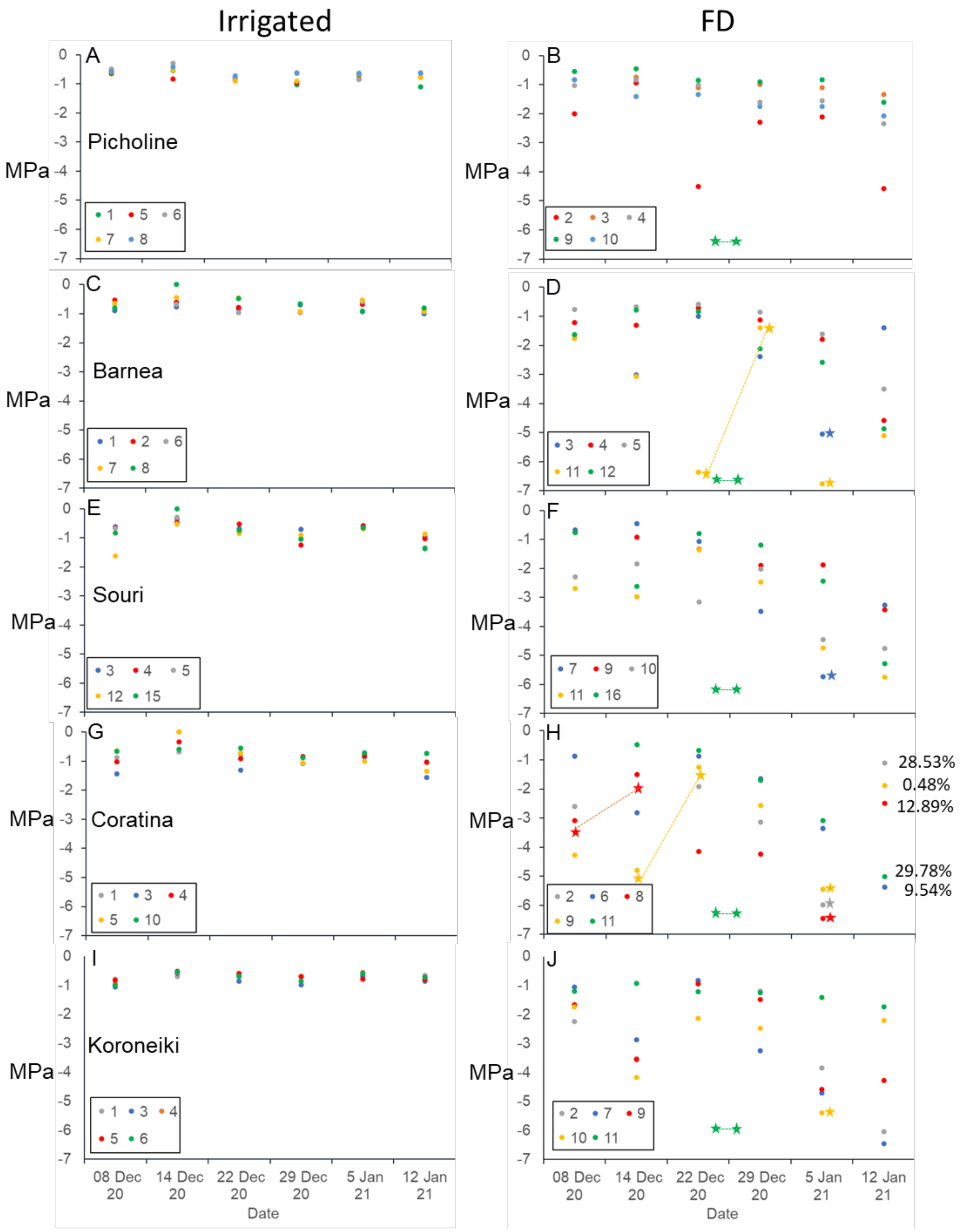

Irrigated
FD
A
Picholine
B
C
Barnea
D
E
Souri
F
G
Coratina
H
I
Koroneiki
J
MPa
Date
28.53%
0.48%
12.89%
29.78%
9.54%

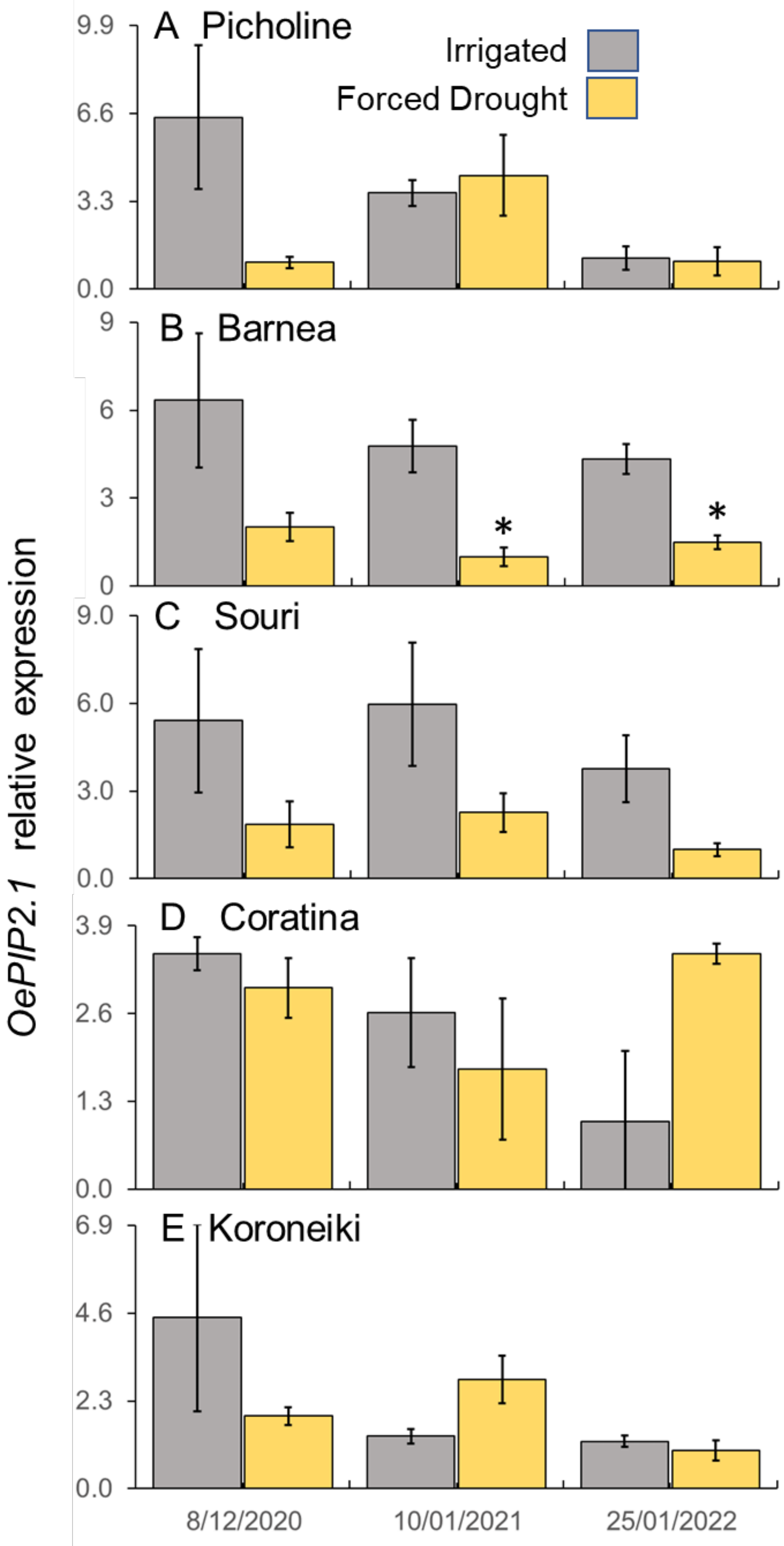

Supplementary Figure 3: Changes in *OePIP2.1* gene expression in different cultivars under forced winter drought. Trees and treatments are described in Figure 2. *OePIP2.1* relative expression in leaves in three-time points during winter, in well-irrigated or Forced Drought trees. Means are made of samples taken from 5 biological repeats (trees). The standard error of the mean is presented as bars. Asterisks denote significant differences P≤0.05 between treatments at the same time point.

Supplementary Figure S4: Changes in *OeFT2* gene expression and percent buds forming inflorescences (*i*) in different cultivars. This figure combines results of well irrigated and FD tress for each cultivar, presented separately in Figure 3. Trees and treatments described in Figure 2. *OeFT2* relative expression in leaves (A), in three time points during winter Means are made of samples taken from 10 biological repeats (trees). Standard error of the mean is presented as bars. *i* values (B) were calculated for 18-20 branches per tree, and the means for each tree calculated. Box and whiskers plot as in Figure 2.

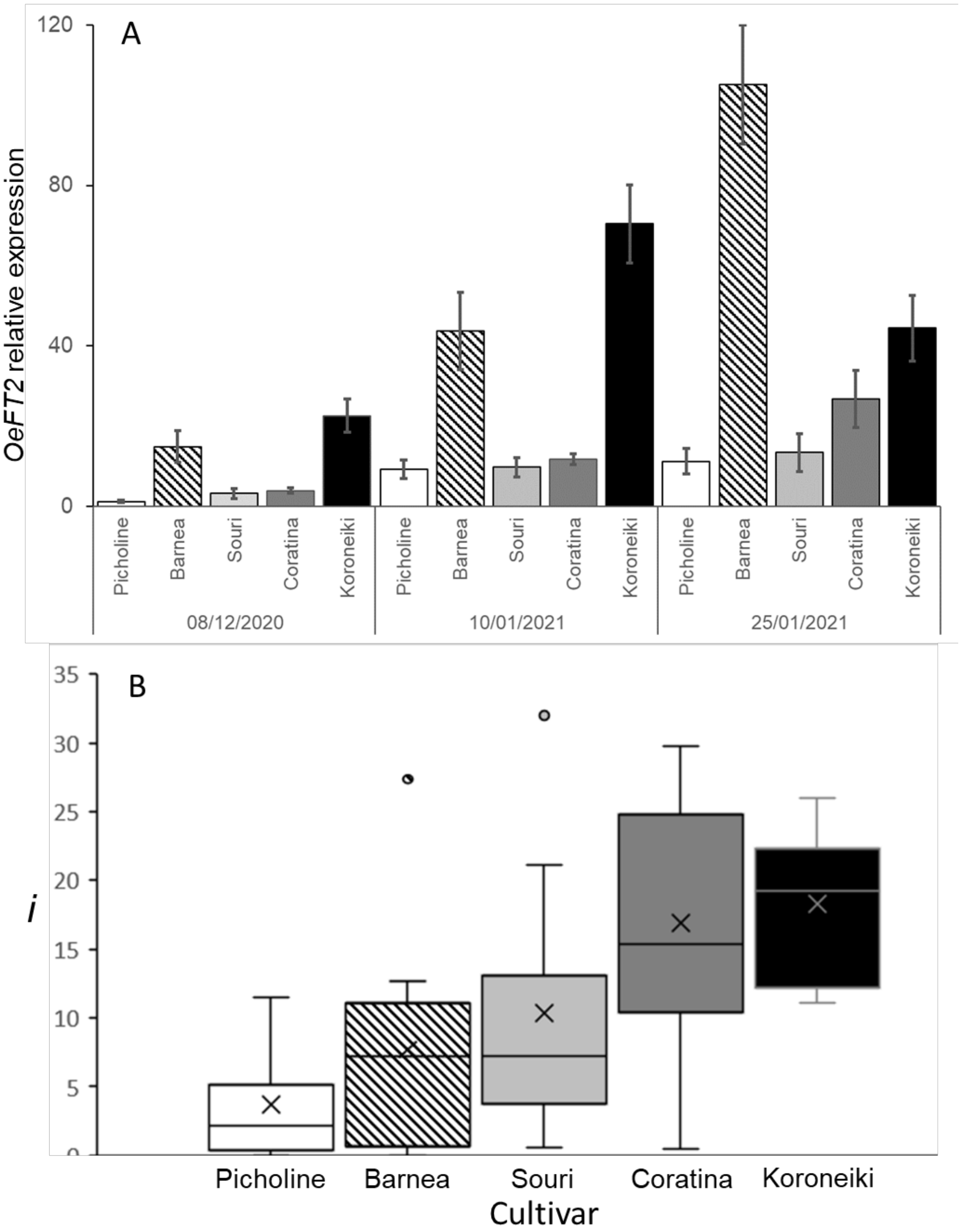

Supplementary Figure S5. Scheme of major experiments presented in the manuscript.

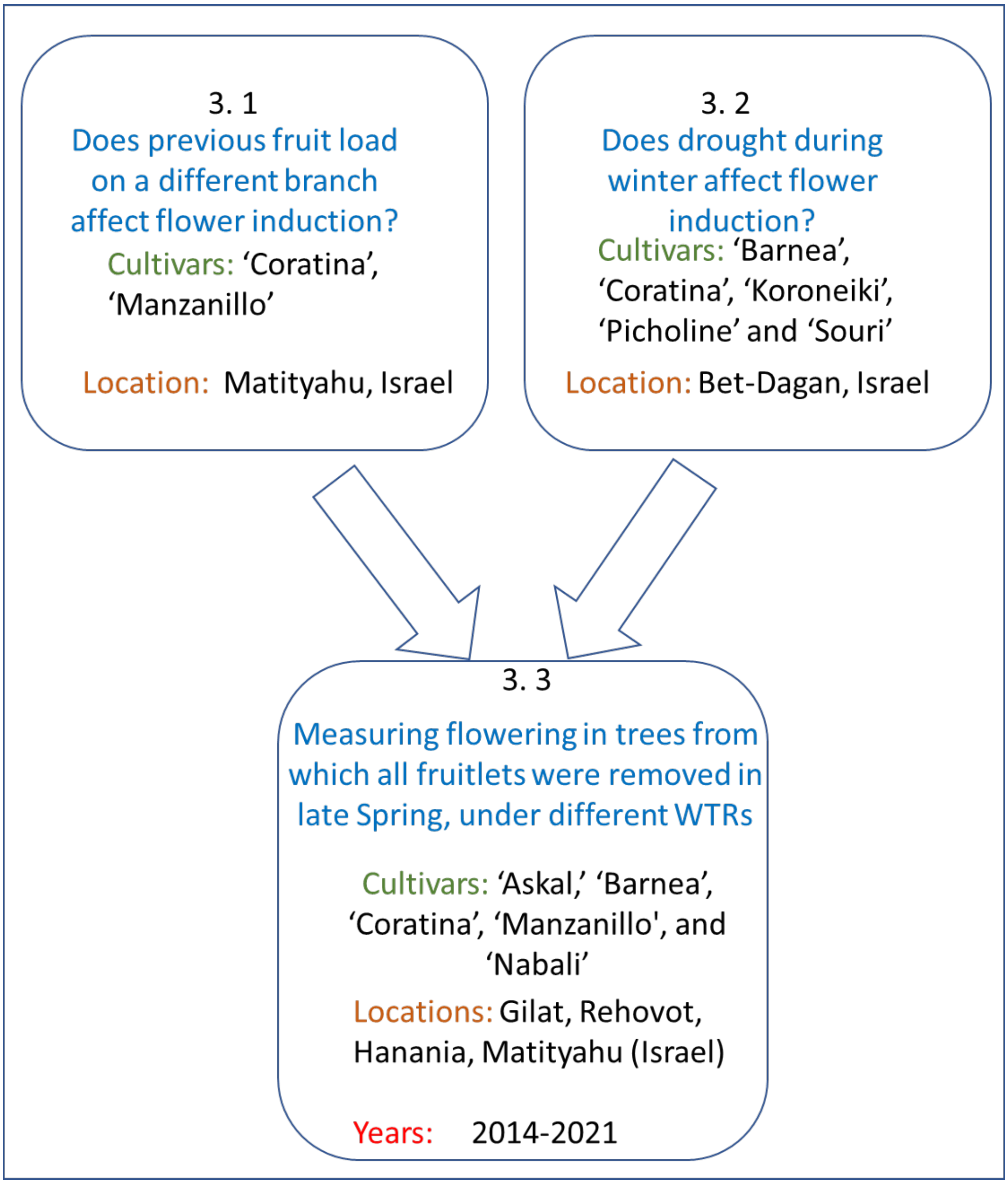

Supplementary Figure S6. Forced Drought (FD) treatment set up. Plant trunks and pots were covered with waterproof cloth and raised on cinderblocks to prevent all water access.

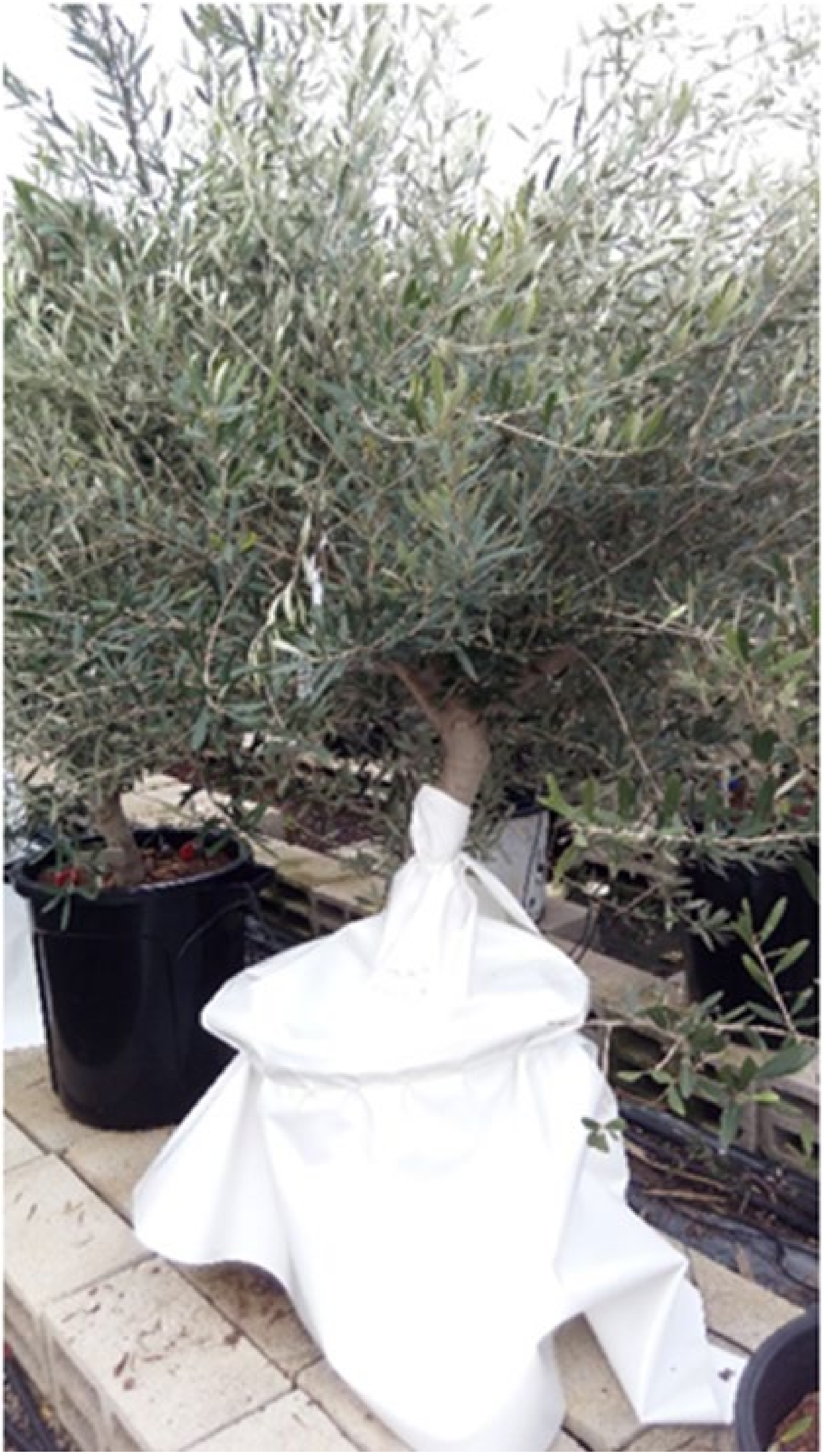

Supplementary Table S1. Effect test of fruit load on the number of buds per branch (*n*)

	DF	Sum of Squares	F Ratio	Prob > F	
**Treatment**	3	88	15.11	6.28E-05	***
**Cultivar**	1	6	3.30	0.0882	
**Treatment*Cultivar**	3	31	5.40	0.0092	**

Supplementary Table S2. Effect test of fruit load on percent buds forming inflorescences (*i*)

	DF	Sum of Squares	F Ratio	Prob > F	
**Treatment**	3	12264	56.20	1.02E-08	***
**Cultivar**	1	23	0.32	0.5784	
**Treatment*Cultivar**	3	3108	14.24	8.81E-05	***

Supplementary Table S3. Effect test of forced drought (FD) on branch water potential (MPA).

	DF	Sum of Squares	F Ratio	Prob > F	
**Cultivar**	4	20	5.48	0.0003	***
**Treatment**	1	192	214.38	6.21E-35	***
**Cultivar*Treatment**	4	14	3.90	0.0044	**
**Date**	5	60	13.37	1.76E-11	***
**Cultivar*Date**	20	20	1.11	0.3431	
**Treatment*Date**	5	51	11.30	8.92E-10	***
**Cultivar*Treatment*Date**	20	19	1.06	0.3893	

Supplementary Table S4. Effect test of forced drought (FD) on *OeFT2* relative expression in leaves.

	DF	Sum of Squares	F Ratio	Prob > F	
**Cultivar**	4	11704	40.33	2.40E-05	***
**Treatment**	1	170	2.35	0.164	
**Cultivar*Treatment**	4	227	0.78	0.5668	
**Date**	2	4892	33.72	0.0001	***
**Cultivar*Date**	8	6309	10.87	0.0014	**
**Treatment*Date**	2	28	0.19	0.8269	

Supplementary Table S5. Effect test of forced drought (FD) on percent buds forming inflorescences (*i*)

	DF	Sum of Squares	F Ratio	Prob > F	
**Cultivar**	4	1659	6.14	0.0006	***
**Treatment**	1	11	0.16	0.6889	
**Cultivar*Treatment**	4	107	0.40	0.8102	

Supplementary Table S6. Flowering data collected after different winters at different locations.

Year	Location	Cultivar				
		Askal	Barnea	Coratina	Manzanillo	Nabali
2014-15	Rehovot	*		*	*	*
2015-16	Rehovot			*	*	*
2016-17	Rehovot					
	Gilat					
	Hanania					
2017-18	Rehovot					
	Rehovot SW**	*		*	*	*
2018-19	Rehovot					
2019-20	Rehovot		*			
	Matityahu					
2020-21	Rehovot					
	Matityahu					

* Data not collected

** Plants moved to a heated glasshouse on December 24th, 2017 until the end of winter.

Supplementary Table S7. Events of temporary irrigation for specific FD trees

Plant	Date of dripper placement	Date of dripper removal	Early end of drought treatment
Coratina 8	Dec 2nd 2020	Dec 14th 2020	Jan 5th 2021
Coratina 9	Dec 14th 2020	Dec 22nd 2020	
Barnea 11	Dec 22nd 2020	Dec 27th 2020	
Barnea 3			
Coratina 2			
Koroneiki 10			
Souri 7			

Supplementary Table S8. Primers for Real-time RTPCR used in this study

Olea europaea			Arabidopsis Reference Protein (from Tair)			Primers	
Name	Accession (transcripts)	Genomic location	Protein Name	Arabidopsis accession	E-value (blastp)	Primer Name	Primer sequence
OeACT7	OE6A117728T1	Oe6_s00163	ACTIN 7 (ACT7)	AT5G09810.1	5.6E-199	OeACT7RTfor	5'-AAGATCAAAGTTGTTGCACCACC-3'
						OeACT7RTrev	5'-CTTAGAAATCCACATCTGCTGGAAT-3'
OeFT2	OE6A103537T1	Oe6_s04126	FLOWERING LOCUS T (FT)	AT1G65480.1	3.8E-74	OeFT2RTfor	5'-CCTTCGTACTTTCTACACGCTCATT-3'
						OeFT2RTrev	5'-TCAGTCACCAACCAGTGCAAA-3'
OePIP2.1	OEA6100469T1	Oe6_s02336	PLASMA MEMBRANE INTRINSIC PROTEIN (PIP2)	AT3G53420.1	4.0E-98	OePIP2RTfor	5'-TTCGTTGCCACTTTGCTGTT -3'
						OePIP2RTrev	5'-TGCTGGGTTAATATGTCCTCCA -3'
